# *In silico* identification of SARS-CoV-2 spike (S) protein–ACE2 complex inhibitors from eight *Tecoma* species and cultivars analyzed by LC-MS†

**DOI:** 10.1039/d0ra08997d

**Published:** 2020-11-26

**Authors:** Seham S. El Hawary, Amira R. Khattab, Hanan S. Marzouk, Amira S. El Senousy, Mariam G. A. Alex, Omar M. Aly, Mohamed Teleb, Usama Ramadan Abdelmohsen

**Affiliations:** Pharmacognosy Department, Faculty of Pharmacy, Cairo University Egypt; Pharmacognosy Department, College of Pharmacy, Arab Academy for Science, Technology and Maritime Transport Alexandria 1029 Egypt Dr_amira_khattab@aast.edu; Pharmacognosy Department, Faculty of Pharmacy, Pharos University in Alexandria Egypt; Medicinal Chemistry Department, Faculty of Pharmacy, Minia University Minia 61519 Egypt; Department of Pharmaceutical Chemistry, Faculty of Pharmacy, Alexandria University Alexandria 21521 Egypt; Pharmacognosy Department, Faculty of Pharmacy, Deraya University Minia 61111 Egypt usama.ramadan@mu.edu.eg; Pharmacognosy Department, Faculty of Pharmacy, Minia University Minia 61519 Egypt

## Abstract

Coronavirus (CoV) is a positive RNA genome virus causing a global panic nowadays. *Tecoma* is a medicinally-valuable genus in the Bignoniaceae family, with some of its species exhibiting anti-HIV activity. This encouraged us to conduct an *in silico* exploration of some phytocompounds in *Tecoma* species cultivated in Egypt, namely *Tecoma capensis* and its four varieties *i.e.* yellow, harmony, pink and red, *T. grandiflora* Loisel., *T. radicans* L., and one hybrid *i.e. Tecoma* × *smithii* W. Watson. LC/MS-based metabolite profiling of the studied *Tecoma* plants resulted in the dereplication of 12 compounds (1–12) belonging to different phytochemical classes *viz.* alkaloids, iridoids, flavonoids and fatty acid esters. The *in silico* inhibitory action of these compounds against SARS-CoV-2 spike protein C-terminal domain in complex with human ACE2 was assessed *via* molecular docking. Succinic acid decyl-3-oxobut-2-yl ester (10), a fatty acid ester, possessed the best binding affinity (−6.77 kcal mol^−1^), as compared to hesperidin (13) (−7.10 kcal mol^−1^).

## Abstract 2

*In silico* exploration of 12 *Tecoma* phytocompounds that could serve as potential inhibitors of SARS-CoV entry to host cells.

## Graphical abstract

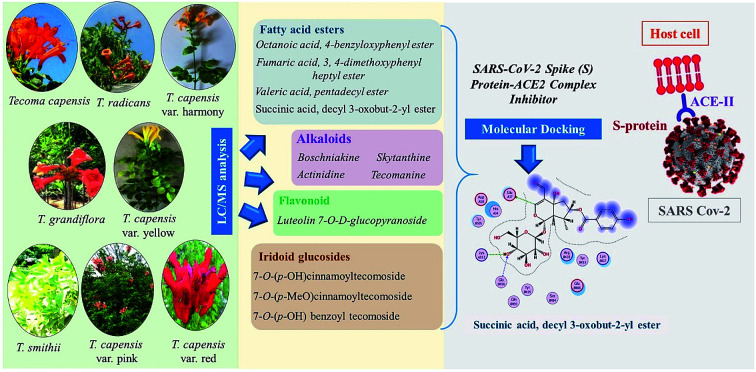

## 1. Introduction

Coronaviruses (CoVs) are positive RNA genome viruses, belonging to the Coronaviridae family of the Nidovirales order, which is divided into four genera (A, B, C and D). SARS-CoV-2 belongs to the B genus. CoV possess four structural proteins: spike protein, envelope protein, membrane protein, and nucleocapsid protein.^1^ Spike protein promotes host attachment and viral cell membrane fusion during virus infection.^2^ Potential anti-coronavirus treatments can be divided into two main categories, one operating on the human immune system or human cells, and the other on the coronavirus itself.^3^ Viruses often bind to receptor proteins on the surface of cells in order to enter human cells, for example, the SARS virus links with human angiotensin-converting enzyme 2 (hACE2) receptor.^4^ Protein–protein docking showed that SARS-CoV-2 spike proteins have a strong affinity for hACE2.^5^ Through virtual screening many compounds could be identified as inhibitors of hACE2, however these potential hACE2 inhibitors might not be beneficial for the management of SARS-CoV-2 infection due to the protective role of hACE2 in lung injury. Hesperidin was the only compound reported till now that could target the binding interface between spike protein and hACE2. It was reported that hesperidin can lie on the middle shallow pit of the surface of the receptor-binding domain (RBD) of spike protein.^6^ Shang J. *et al.*, determined the crystal structure of RBD of the spike protein of SARS-CoV-2 (engineered to facilitate crystallization) in complex with hACE2. In comparison with the SARS-CoV RBD, an hACE2-binding ridge in SARS-CoV-2 RBD has a more compact conformation; moreover, several residues changes in the SARS-CoV-2 RBD stabilize two virus-binding hotspots at the RBD–hACE2 interface. These structural features of SARS-CoV-2 RBD increase its hACE2 binding affinity.^7^

*Tecoma* genus is one of the medicinally-valuable members in Bignoniaceae family, embracing fourteen species of shrubs and small trees, of which twelve are native to America and two to Africa. The leaves were traditionally used by people in Latin America for diabetes management.^8^ Later on, several *Tecoma* species were reported to possess a wide variety of pharmacological actions *viz.* anti-inflammatory, antipyretic, analgesic, antimicrobial, antioxidant, hepatoprotective and cytotoxic actions.^9^*Tecoma stans* (L.) Juss. ex Kunth, *Tecoma capensis* and *Tecoma undulata* possessed anti-diabetic action. *T. sambucifolia* H.B.K. had anti-inflammatory and antinociceptive actions as well as cytotoxicity against human hepatoma cell line. *T. undulata* Seem. showed hepatoprotective actions.^10^*Tecoma* species are reported to be enriched in a diverse array of phytochemicals *viz.* alkaloids, flavonoids, iridoids, naphthoquinones, coumarins, chromones and steroids.^10^

Many research endeavors are now directed towards finding an effective therapy against COVID 19.^11–18^ As an extension from our previous research work,^2,19,20^ we conducted a screening for natural products that could possess potential anti-SARS actions, however with more focus on the plants reported to possess antiviral as well as antimalarial activities (compared to the antimalarial medication “chloroquine” with approved anti SARS-CoV activity^21^). We found that among *Tecoma* species, *T. undulata* was reported to exhibit anti-HIV activity^22^ and *T. mollis* possessed anti-malarial activity using chloroquine sensitive clones of *Plasmodium falciparum*.^23^ These reports encouraged us to conduct an *in silico* exploration of some representative members of *Tecoma* species cultivated in Egypt namely *i.e. Tecoma capensis* Lindl., *T. capensis* var. yellow, *T. capensis* var. harmony, *T. grandiflora* Loisel., *T. radicans* (L.) Juss., *T. capensis* var. pink, *T. capensis* var. red, and one hybrid *Tecoma* × *smithii* W. Watson.

## 2. Materials and methods

### 2.1. Plant material

Fresh samples of eight *Tecoma* species and cultivars were collected during the years 2016 and 2017, which are enlisted in ESI (Table S1)† along with their collection geographical locations and voucher specimen codes. The botanical specimens were identified by Professor Selim Zidan (Head of Botany Department, Faculty of Science, Alexandria University, Egypt), Mrs Therese Labib (Mazhar Botanical Garden, Egypt) and Mr Mohamed El-Gebaly (Plant Taxonomists in El-Orman Botanical Garden, Egypt).

### 2.2. Preparation of plant extract and LC-MS metabolic profiling

Leaves of *Tecoma* species and cultivars under study (1600 g) were exhaustively extracted with methanol by cold maceration in 10 L methanol (2 L each × 5 times). The total methanolic extract was evaporated under reduced pressure by rotary evaporator at a temperature not exceeding 45 °C, yielding 180–200 mg of total methanolic extract of each studied plants. About 2 mg of each crude methanolic extract was dissolved separately in 1 ml MeOH and filtered using 0.2 μm membrane filter and then subjected to LC-HRESIMS analysis as previously reported in ref. 24. An Acquity Ultra-Performance Liquid Chromatography (UPLC) system coupled to a Synapt G2 HDMS quadrupole time-of-flight hybrid mass spectrometer (Waters, Milford, MA, USA). Chromatographic separation was performed using a BEH C18 column (2.1 × 100 mm, 1.7 mm particle size) and a guard column (2.1 × 5 mm, 1.7 mm particle size) using the method previously described in ref. 19. Fig. S1† depicts the total ion chromatograms of the eight studied plants. MZmine 2.12 was used for processing the obtained raw data by the negative ionization mode. The processed data set was then subjected to molecular formula prediction and peak identification *via* dereplication using online METLIN^25^ and Dictionary of Natural Products (DNP)^26^ databases.

### 2.3. Docking studies

#### 2.3.1. Docking of the target phytocompounds

Molecular docking study was carried out using Molecular Operating Environment (MOE) software package version 2016.10, Chemical Computing Group, Montreal, Canada. The crystal structure of SARS-CoV-2 spike (S) protein C-terminal domain (SARS-CoV-2-CTD) in complex with human ACE2 (hACE2) was obtained from the protein data bank (PDB ID 6LZG).^27^ Unwanted residues, ligands and solvents were eliminated, then the complex was prepared employing the default “Structure preparation” module settings. ‘Site Finder’ feature of MOE 2016.10 was employed in search for the receptor site in the SARS-CoV-2-CTD binding interface. MDB database file of the target phytocompounds was subjected to default energy minimization and geometry optimization. Triangular matcher algorithm was applied to set the ligand placement. The default scoring function was alpha HB which generated the top 5 non-redundant poses of the lowest binding energy conformers of the tested phytoligands. Docking was conducted with induced fitting protocol to record the best possible molecular interactions. Results were listed based on the *S*-scores with RMSD value < 2 Å. Graphical representations of the phytoligands interactions were then generated and inspected.

## 3. Results and discussion

### 3.1. LC-HRESIMS based metabolic profiling

Twelve phytocompounds belonging to different phytochemical classes *viz.* alkaloids, iridoids, flavonoids, and fatty acid esters, have been identified by dereplication of the obtained LC-HRESIMS derived metabolite profiles of the eight *Tecoma* species and cultivars (Table 1). These metabolites have been annotated using databases (METLIN and DNP databases). From these databases, the molecular ion peaks appeared at *m*/*z* 325.1835, 349.1141, 327.2163 and 311.1672, with their predicted molecular formulas of C_21_H_26_O_3_, C_19_H_26_O_6_, C_18_H_32_O_5_ and C_20_H_40_O_2_ were dereplicated to be corresponding to four fatty acid esters *viz.* octanoic acid, 4-benzyloxyphenyl ester (1), fumaric acid, 3,4-dimethoxyphenyl heptyl ester (2), succinic acid, decyl 3-oxobut-2-yl ester (10) and valeric acid, pentadecyl ester (12), respectively.

**Table 1:** List of the identified metabolites in leaf methanolic extracts of 8 *Tecoma* plants under study using LC-HRESIMS

#	Compound name	*R*_T_ (min)	[M − H]^−^	Molecular formula	Error (0.1 *m*/*z* or 5 ppm)	*T. smithii* Wil. Wats.	*T. radicans* (L.) Juss.	*T. capensis* var. harmony	*T. capensis* var. yellow	*T. capensis* var. pink	*T. capensis* var. red	*T. grandiflora* (Thunb.) Loisel.	*T. capensis* (Thunb.) Lindl.
1	Octanoic acid, 4-benzyloxyphenyl ester	1.32	325.1835	C_21_H_26_O_3_	0.0134	+	−	+	+	−	+	+	+
2	Fumaric acid, 3,4-dimethoxyphenyl heptyl ester	1.82	349.1141	C_19_H_26_O_6_	−0.0495	+	+	−	+	−	+	+	+
3	Boschniakine	2.07	160.5217	C_10_H_11_NO	0.0446	+	+	+	+	+	−	−	+
4	Luteolin 7-*O*-d-glucopyranoside	2.18	447.1537	C_21_H_20_O_11_	0.0604	−	−	−	+	+	−	−	+
5	Actinidine	2.89	147.9783	C_10_H_13_N	0.0109	+	+	+	+	+	−	+	+
6	Skytanthine	3.16	166.3757	C_11_H_21_N	0.02156	+	−	+	+	+	+	+	+
7	Tecomanine (syn. tecomine)	3.35	178.0705	C_11_H_17_NO	0.0013	+	−	−	+	+	−	+	+
8	7-*O*-(*p*-OH)cinnamoyltecomoside	3.38	521.1938	C_25_H_30_O_12_	0.0973	−	−	+	+	−	+	−	+
9	7-*O*-(*p*-MeO)cinnamoyltecomoside	3.60	535.2093	C_26_H_32_O_12_	0.0122	−	−	+	+	+	+	−	+
10	Succinic acid, decyl 3-oxobut-2-yl ester	3.78	327.2163	C_18_H_32_O_5_	0.0134	+	−	+	+	−	+	+	+
11	7-*O*-(*p*-OH)benzoyltecomoside	4.96	495.2212	C_23_H_28_O_12_	0.0267	−	−	+	+	+	−	−	−
12	Valeric acid, pentadecyl ester	6.78	311.1672	C_20_H_40_O_2_	0.0537	+	+	−	+	−	+	+	+

Besides, four alkaloids were dereplicated; boschniakine (3) with its molecular ion peak detected at *m*/*z* 160.5217, and a predicted molecular formula of C_10_H_11_NO, which was formerly identified in *T. stans*.^28,29^ Besides, actinidine (5), skytanthine (6), and tecomanine (7) were identified based on their molecular ion peaks appearing at *m*/*z* 147.9783, 166.3757 and 178.0705 and possessing predicted molecular formulas of C_10_H_13_N, C_11_H_21_N and C_11_H_17_NO which were previously isolated from several *Tecoma* spp. *i.e. T. stans* and *T. arequipensis*.^28,30^ Three iridoid glucosides were tentatively identified as being 7-*O*-(*p*-OH)cinnamoyltecomoside (8), 7-*O*-(*p*-MeO)cinnamoyltecomoside (9) and 7-*O*-(*p*-OH)benzoyl tecomoside (11) based on their respective molecular ion peaks at *m*/*z* 521.1938, 535.2093 and 495.2212, and molecular formulas of C_25_H_30_O_12_, C_26_H_32_O_12_ and C_23_H_28_O_12_. These compounds were previously isolated from *T. capensis*,^31^ however, they are reported here in most of the studied *Tecoma* species for the first time. One flavonoid was identified as being luteolin 7-*O*-β-d-glucopyranoside (4) by its molecular ion peak depicted at *m*/*z* 447.1537, corresponding to the suggested molecular formula (C_21_H_20_O_11_) which was formerly detected in *T. stans* fruits.^32^

### 3.2. Molecular docking analysis of selected phytocompounds from *Tecoma* species and cultivars

Docking simulations results (Table 2) showed that most of the studied phytocompounds displayed moderate to promising binding affinities compared to hesperidin (13). Succinic acid, decyl-3-oxobut-2-yl ester (10) came at the top of the list due to its best binding affinity (−6.77 kcal mol^−1^) among the studied phytocompounds. 7-*O*-(*p*-OH)cinnamoyltecomoside (8), valeric acid, pentadecyl ester (12), octanoic acid, 4-benzyloxyphenyl ester (1), 7-*O*-(*p*-OH)benzoyl tecomoside (11) showed slightly less binding affinities ranging from −6.73 to −6.48 kcal mol^−1^. Moderate fitting was observed in case of the two phytocompounds *viz.* 7-*O*-(*p*-MeO)cinnamoyltecomoside (9), and fumaric acid, 3,4-dimethoxyphenyl heptyl ester (2) with binding affinities of −5.44 and −5.28 kcal mol^−1^, respectively. The remaining phytocompounds showed relatively low binding affinities.

**Table 2:** Docking simulations results of the studied *Tecoma* phytocompounds

No.	Name of phytoligands	Δ*G*^a^ (kcal mol^−1^)	Interactions at the binding interface^b^	
			hACE2 residues	SARSCoV-2-CTD residues
1	Octanoic acid, 4-benzyloxyphenyl ester	−6.66	No interaction	Gln493
2	Fumaric acid, 3,4-dimethoxyphenyl heptyl ester	−5.28	No interaction	Glu406, Arg408, **Lys417**
3	Boschniakine	−3.76	**Lys353**	Gln493
4	Luteolin 7-*O*-d-glucopyranoside	−4.84	Glu37, **Lys353**	Glu406, Gln493
5	Actinidine	−4.80	No interaction	No interaction
6	Skytanthine	−4.47	No interaction	Arg403
7	Tecomanine (syn. tecomine)	−4.64	**Lys353**	**Gly496**
8	7-*O*-(*p*-OH)cinnamoyltecomoside	−6.73	No interaction	Glu406, Gln409
9	7-*O*-(*p*-MeO)cinnamoyltecomoside	−5.44	**His34**, Ala389, Arg393	Arg403
10	Succinic acid, decyl 3-oxobut-2-yl ester	−6.77	**His34**, Glu35	Gln493
11	7-*O*-(*p*-OH)benzoyl tecomoside	−6.48	Glu37, **Lys353**	Asp405, Arg408, **Gly496**
12	Valeric acid, pentadecyl ester	−6.73	**Lys353**	**Gly496**
13	Hesperidin	−7.10	**His34**, Ala387	Gln409, **Lys417**, Ser494

a The ligand–receptor complex binding free energy at RMSD < 2 Å.

b The key residues involved in the SARS-CoV-2-CTD–2hACE complex formation are listed in bold.

**Fig. 1:**
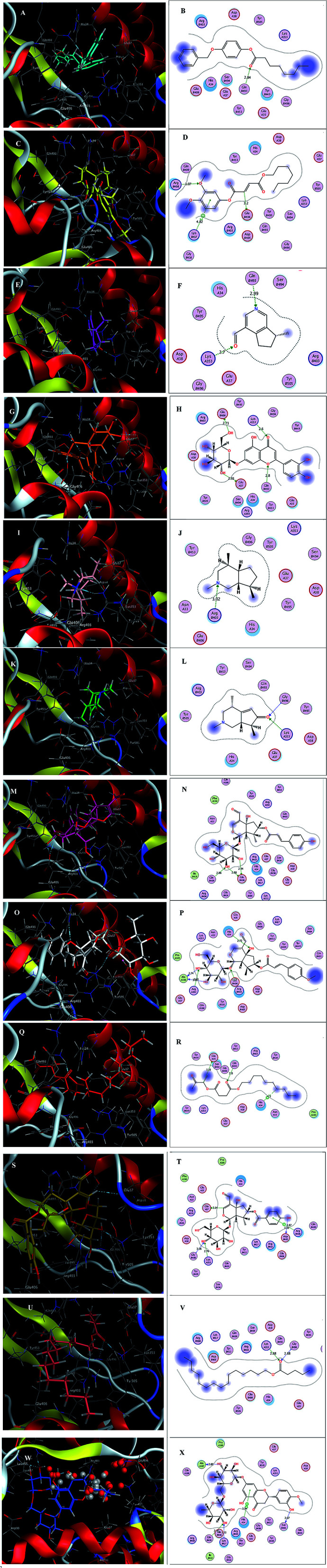
(A) 3D binding mode of 1 (cyan sticks), (B) 2D binding mode of 1, (C) 3D binding mode of 2 (yellow sticks), (D) 2D binding mode of 2, (E) 3D binding mode of 3 (magenta sticks), (F) 2D binding mode of 3, (G) 3D binding mode of 4 (orange sticks), (H) 2D binding mode of 4, (I) 3D binding mode of 6 (light pink sticks), (J) 2D binding mode of 6, (K) 3D binding mode of 7 (green sticks), (L) 2D binding mode of 7, (M) 3D binding mode of 8 (deep pink sticks), (N) 2D binding mode of 8, (O) 3D binding mode of 9 (white sticks), (P) 2D binding mode of 9, (Q) 3D binding mode of 10 (red sticks), (R) 2D binding mode of 10, (S) 3D binding mode of 11 (deep yellow sticks), (T) 2D binding mode of 11, (U) 3D binding mode of 12 (pink sticks), (V) 2D binding mode of 12 (W) 3D binding mode of 13 (blue sticks), (X) 2D binding mode of 13 in the binding interface of SARS-CoV-2-CTD in complex with hACE2 (PDB ID 6LZG).

**Fig. 2:**
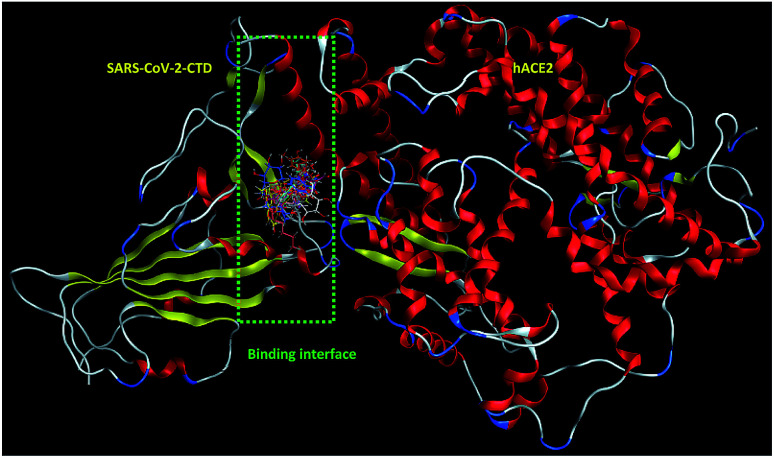
Overlay of 1 (cyan sticks), 2 (yellow sticks), 3 (magenta sticks), 4 (orange sticks), 6 (light pink sticks), 7 (green sticks), 8 (deep pink sticks), 9 (white sticks), 10 (red sticks), 11 (deep yellow sticks), 12 (pink sticks) and 13 (blue sticks) in the binding interface of SARS-CoV-2-CTD in complex with hACE2 (PDB ID 6LZG).

Inspecting the binding modes of the promising phytocompounds revealed that most of them were able to accommodate into the interface and interact with the key amino acids, in most cases, through hydrogen bonds (Fig. 1 and 2). Thus, they may destabilize or even prevent the virus–receptor engagement that is generally dominated by polar contacts mediated by these key hydrophilic amino acid residues.^27^ Succinic acid, decyl-3-oxobut-2-yl ester (10), showing the best binding affinity in the current study, exhibited H–π interactions with His34 of the hACE-2 involved in the complex formation (Fig. 1Q and R). Among the studied phytocompounds, the second most promising *in silico* activity was recorded by valeric acid, pentadecyl ester (12) which interacted with SARS-CoV-2-CTD Gly496 and hACE2 Lys353 (Fig. 1U and V). Similarly, 7-*O*-(*p*-OH)benzoyl tecomoside (11) exhibited these key interactions in addition to binding hACE-2 Glu37 (Fig. 1S and T). It is worth mentioning that a single Lys353 mutation was reported to be sufficient to abolish the interactions at the interface. Interestingly, boschniakine (3; Fig. 1E and F), luteolin 7-*O*-d-glucopyranoside (4; Fig. 1G and H), tecomanine (7; Fig. 1K and L) were able to interact with Lys353 despite their relatively low binding affinities to the complex. Notably, fumaric acid, 3,4-dimethoxyphenyl heptyl ester (2) showed interactions with SARS-CoV-2 CTD through its key residue Lys417 as well as Glu406 with no interactions with the hACE-2 side (Fig. 1C and D). This observation was also recorded in case of octanoic acid, 4-benzyloxyphenyl ester (1; Fig. 1A and B), skytanthine (6; Fig. 1I and J) and 7-*O*-(*p*-OH)cinnamoyltecomoside (8; Fig. 1M and N). On the other hand, actinidine (5) did not exhibit any considerable interactions with its residues.

## 4. Conclusions

In the current study, *in silico* exploration was conducted for some of *Tecoma* phytocompounds that could inhibit SARS-CoV entry to its host cells. Twelve compounds from eight *Tecoma* plants belonging to different phytochemical classes *viz.* alkaloids, iridoids, flavonoids, and fatty acid esters were dereplicated using LC-HRESIMS, among which a fatty acid ester named succinic acid, decyl-3-oxobut-2-yl ester (10) was reported to possess the best binding affinity (−6.77 kcal mol^−1^), followed by 7-*O*-(*p*-OH)cinnamoyltecomoside (8), valeric acid, pentadecyl ester (12), octanoic acid, 4-benzyloxyphenyl ester (1), 7-*O*-(*p*-OH)benzoyl tecomoside (11) which showed slightly less binding affinities ranging from −6.73 to −6.48 kcal mol^−1^, as compared to hesperidin (13). These phytocompounds could serve as potential candidates for the discovery of anti-SARS drugs that possess a preventive potential, however the therapeutic potential is yet to be validated using *in vitro* and *in vivo* studies.

## Conflicts of interest

The authors declare no conflict of interest.

## Supplementary Material

RA-010-D0RA08997D-s001
