# Supplementary material for: In silico identification of SARS-CoV-2 spike (S) protein–ACE2 complex inhibitors from eight Tecoma species and cultivars analyzed by LC-MS
Source: RSC Adv. 2020 Nov 26;10(70):43103–8. doi: 10.1039/d0ra08997d (PMC9058143; doi:10.1039/d0ra08997d)
Supplement: RA-010-D0RA08997D-s001 [file RA-010-D0RA08997D-s001.pdf]

Electronic Supplementary Material (ESI) for RSC Advances.
This journal is © The Royal Society of Chemistry 2020

Supplementary material

Table S1: List of *Tecoma* species and cultivars under study along with their collection areas and voucher specimen codes.

**Plant name**	**Collection area**	**Voucher specimen codes**
Tecoma capensis Lindl.	St. Mina monastery garden, King Mariot, Borg El Arab, Alexandria, Egypt	No. 11-1-2017I
T. capensis var. yellow	Mazhar Botanical garden, 26th July corridor, Imbaba, Giza, Egypt	No. 11-1-2017II
T. capensis var. harmony	Mazhar Botanical garden, 26th July corridor, Imbaba, Giza, Egypt	No. 11-1-2017III
T. grandiflora Loisel.	Mazhar Botanical garden, 26th July corridor, Imbaba, Giza, Egypt	No. 11-1-2017IV
T. radicans (L.) Juss.	Antonyades Botanical Garden, Alexandria, Egypt	No. 11-1-2017V
T. capensis var. pink	Mazhar Botanical garden, 26th July corridor, Imbaba, Giza, Egypt	No. 11-1-2017VI
T. capensis var. red	Mazhar Botanical garden, 26th July corridor, Imbaba, Giza, Egypt	No. 11-1-2017VII
Tecoma ×smithii W. Watson	El-Orman Botanical garden, Giza, Egypt.	No. 11-1-2017VIII

Figure S1: Total ion chromatograms of leaf methanolic extracts of (a) *T. grandiflora*, (b) *T. smithii,* (c) *T. capensis* var. pink, (d) *T. radicans*, (e) *T. capensis* (Thunb.) lindl, (f) *T. capensis* var. harmony, (g) *T. capensis* var. red, (h) *T. capensis* var. yellow.

(a)

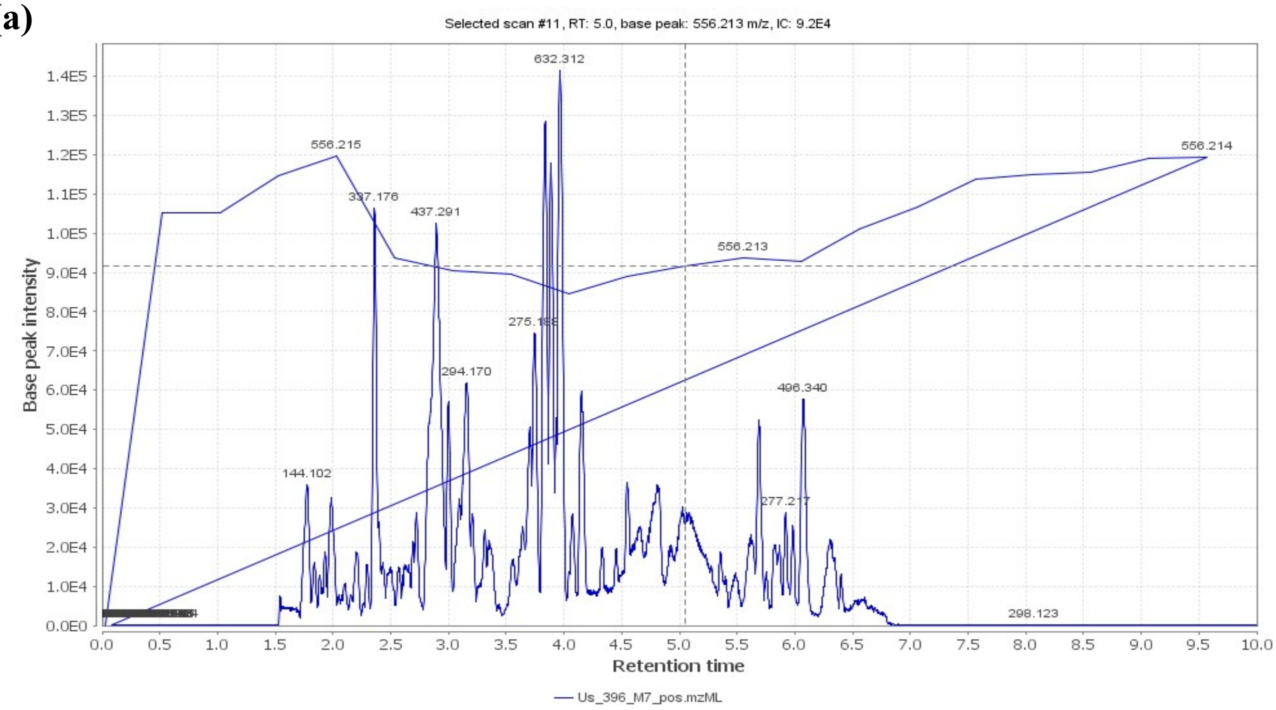

(b)

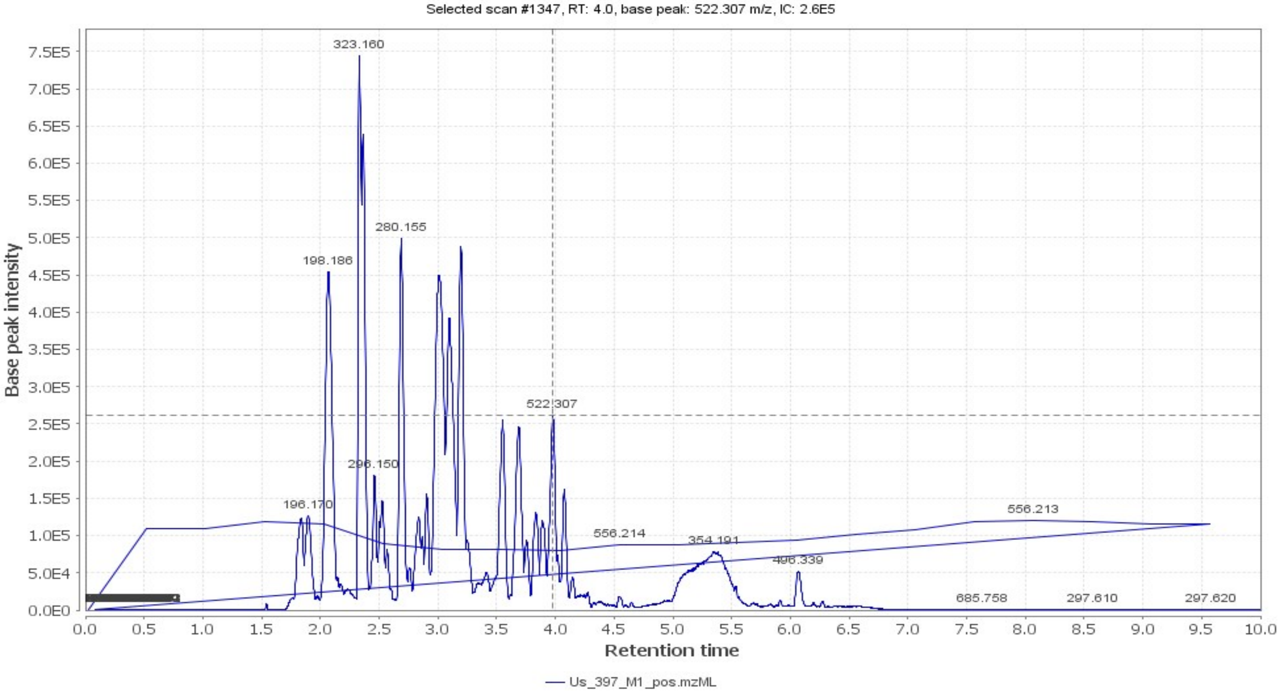

(c)

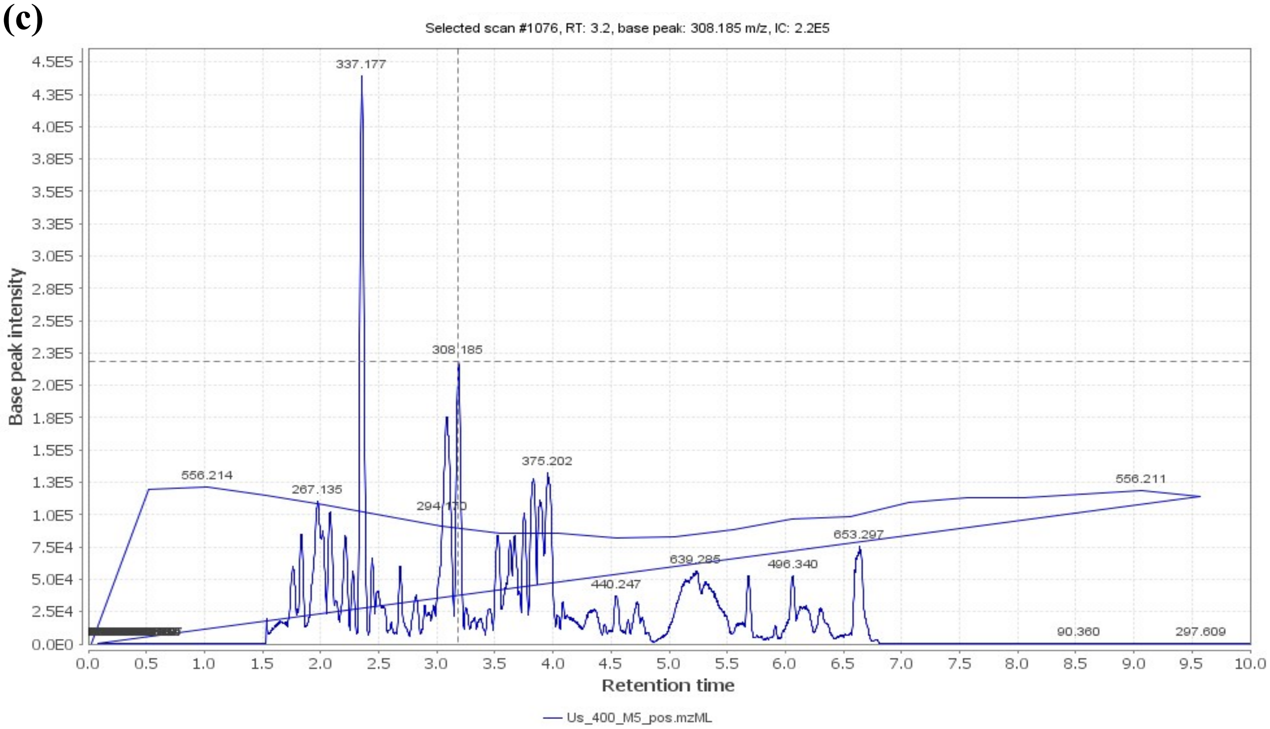
Selected scan #1076, RT: 3.2, base peak: 308.185 m/z, IC: 2.2E5
Base peak intensity
Retention time
337.177
308.185
556.214
267.135
294.170
375.202
556.211
653.297
639.285
496.340
440.247
90.360
297.609
Us_400_M5_pos.mzML

(d)

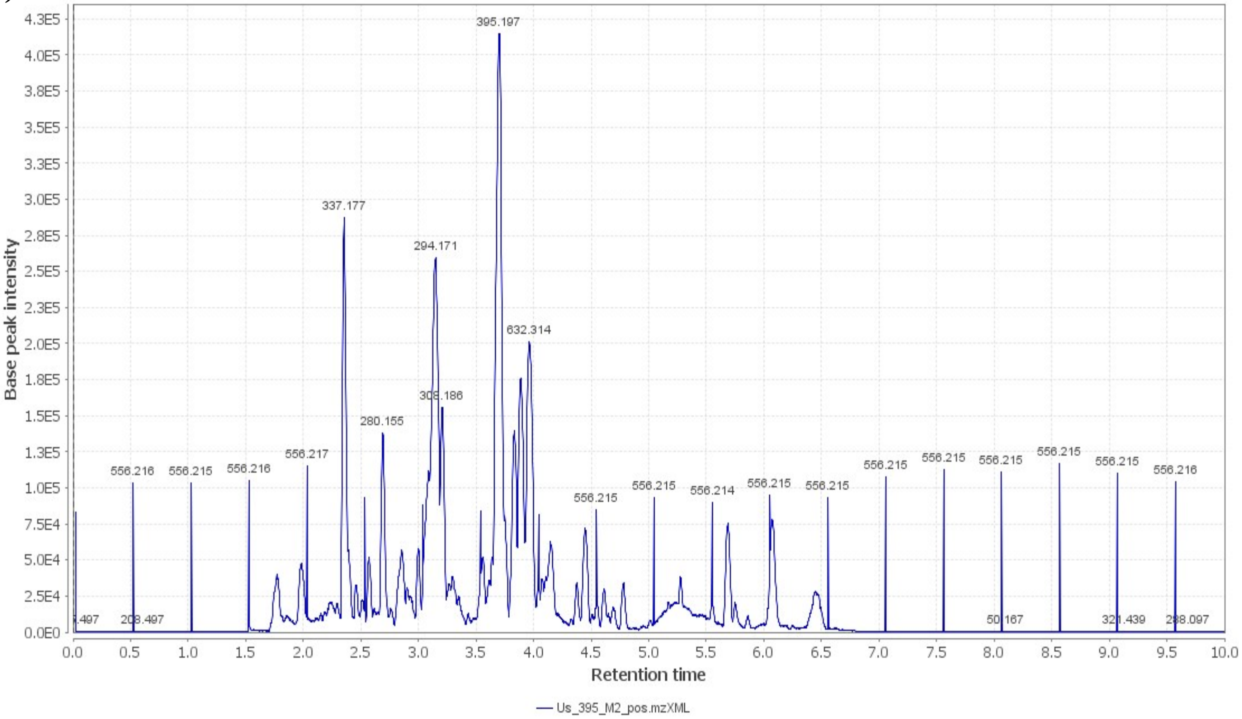
Base peak intensity
Retention time
395.197
337.177
294.171
632.314
308.186
280.155
556.217
556.216
556.215
556.216
556.215
556.215
556.214
556.215
556.215
556.215
556.215
556.215
556.215
556.215
556.216
208.497
50.167
321.439
288.097
Us_395_M2_pos.mzXML

(e)

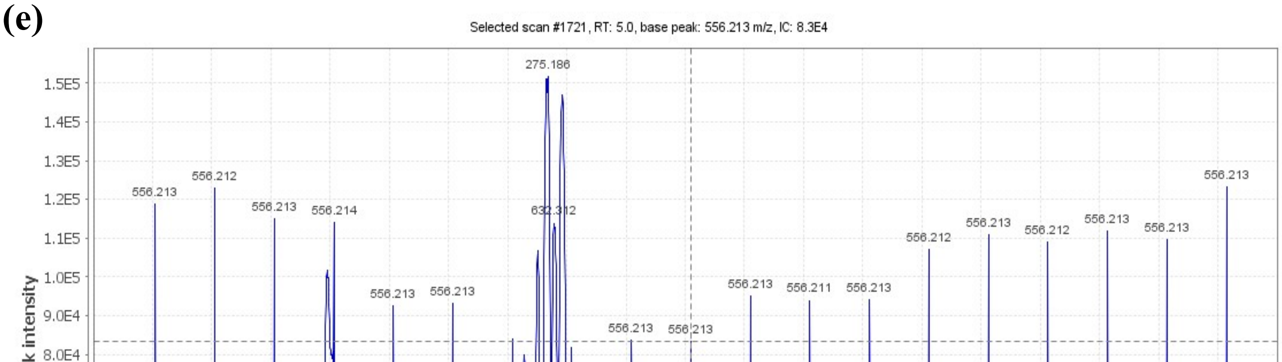
Selected scan #1721, RT: 5.0, base peak: 556.213 m/z, IC: 8.3E4
275.186
632.312
556.213
556.212
556.213
556.214
556.213
556.213
556.213
556.213
556.213
556.211
556.213
556.212
556.213
556.212
556.213
556.213
556.213

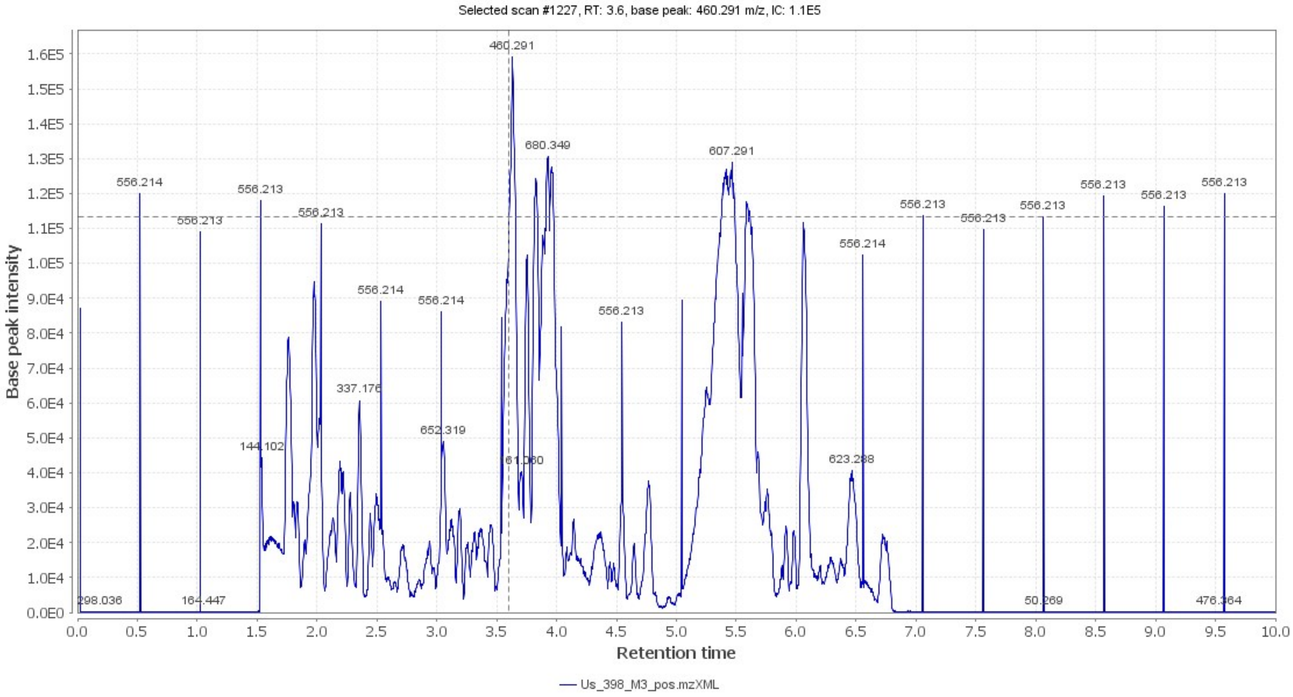
Selected scan #1227, RT: 3.6, base peak: 460.291 m/z, IC: 1.1E5
Base peak intensity
Retention time
Us_398_M3_pos.mzXML

(g)

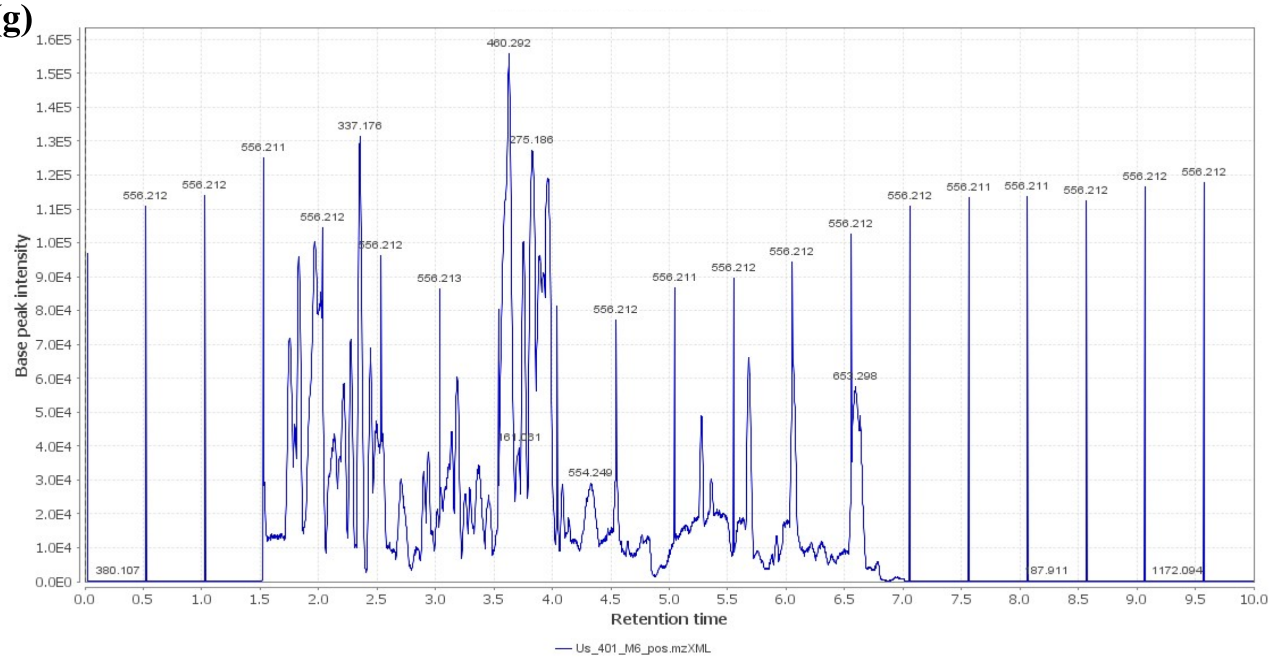
Base peak intensity
Retention time
Us_401_M6_pos.mzXML

(h)

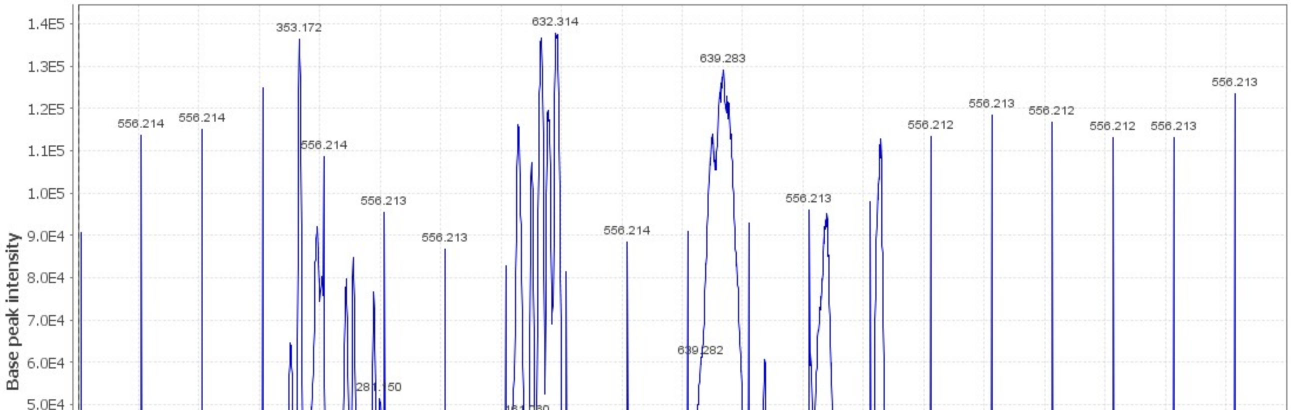
Base peak intensity
